# Juvenile justice systems of care: results of a national survey of community supervision agencies and behavioral health providers on services provision and cross-system interactions

**DOI:** 10.1186/s40352-019-0093-x

**Published:** 2019-06-14

**Authors:** Christy K. Scott, Michael L. Dennis, Christine E. Grella, Rodney R. Funk, Arthur J. Lurigio

**Affiliations:** 1 Chestnut Health Systems, 221 W. Walton St., Chicago, IL 60610 USA; 2 Loyola University Chicago, Chicago, USA

**Keywords:** Adolescents, Community supervision, Juvenile justice, Behavioral health, Substance use

## Abstract

**Background:**

Youth involved in the juvenile justice (JJ) system have high needs for behavioral health services, especially related to substance use and mental disorders. This study aimed to understand the extent to which elements in the cascade model of behavioral health services for JJ-involved youth are provided to youth by Community Supervision (CS) and/or Behavioral Health (BH) providers. In order to understand interactions across CS and BH systems, this study used a multistage probabilistic survey design to sample CS agencies and their primary BH service providers of substance use and mental health treatment in the United States. Parallel surveys were administered to both CS and BH providers regarding: characteristics of youth served, BH services available, whether services were provided directly and/or by referral, use of evidence-based practices (EBPs), and methods of collaboration, referral, and information exchange across CS and BH providers.

**Results:**

The findings from weighted national estimates demonstrate that youth referred from CS to the BH programs represent a more severe sub-group of youth under CS supervision. There are established cross-system relationships for assessment and referral for substance use and mental health treatment, but less so for prevention services. Most CS programs refer youth to BH providers for these services, which typically utilize more highly trained staff to provide EBPs to a majority of the youth served. More intensive substance use and mental health treatment, aftercare, and recovery support services were limited in availability.

**Conclusions:**

The findings suggest that although many elements in a cascade model of BH services for JJ-involved youth have been implemented within local systems of care through collaboration between CS and BH providers, there are several underdeveloped areas and potential for attrition across the service cascade. Greater attention to providing services to youth with higher levels of severity, aftercare services, and recovery support is warranted within a multi-systemic framework.

**Electronic supplementary material:**

The online version of this article (10.1186/s40352-019-0093-x) contains supplementary material, which is available to authorized users.

This article examines the types and extent of collaborations among community supervision (CS) and behavioral health (BH) service providers to youth involved in the juvenile justice system (JJS) within local community-based systems of care. Although CS is the most common dispositional alternative in the JJS (Kaeble & Glaze, 2016), it is also one of the least studied in terms of its actual practices (Willison, Mears, Schollenberger, Owens, & Butts, 2009). Community supervision is an umbrella term that includes court supervision, probation and parole (Champion, 2001); most CS agencies also manage youth who have been assigned deferred adjudication or diversion statuses. In addition, CS agencies set prevailing policies, enact supervisory protocols, and establish linkages with BH providers. As the central component of the JJS, it is important to understand the service needs of youth under CS and types of services provided to them to address their substance use and associated BH problems.

## Background

### Behavioral health problems among youth in the JJS

Youth involved in the JJS exhibit a higher prevalence of BH problems compared with their non-JJS-involved counterparts (Abram et al., 2003; Grisso, 2004). For example, in the United States, an estimated 45% to 65% of youth in the JJS meet criteria for having a substance use disorder (Dennis et al. 2009; Teplin et al. 2002; Timmons-Mitchell et al. 1997). Teplin et al. (2002) conducted clinical assessments with a random sample of youth from the Cook County Juvenile Temporary Detention Center. Nearly two thirds of the males and three quarters of females met criteria for at least one psychiatric disorder; and approximately half of both males and females had a substance use disorder. Fewer than half of these adolescents receive any substance use services, and fewer than one-third of them are treated for a substance use disorder while on community supervision (e.g., juvenile probation and parole; Dennis et al. 2009; Shufelt & Cocozza, 2006; Teplin et al. 2002; Wasserman et al. 2002).

In addition, JJS-involved youth typically report histories of trauma and victimization (Abram et al., 2004, 2007; Ford et al., 2013), childhood maltreatment (King et al., 2011), suicidality (Abram et al., 2008; Tapia et al., 2016; Teplin et al., 2015), and self-injury (Chapman & Ford, 2008; Ford et al. 2010). JJS-involved youth are vulnerable to the human immunodeficiency virus (HIV) and other sexually transmitted infections (STIs) because of their risky sexual activities (Donenberg et al. 2015). The high rates of substance use and other psychiatric disorders among this population contribute to unhealthy sexual behaviors that further increase their risk of contracting HIV and other STIs (Teplin et al. 2003; Romero et al., 2007). Many JJ-involved youth also suffer from cognitive deficits and poor intellectual functioning, highlighting a need to design services for JJS-involved youth that take into consideration their intellectual challenges (Lansing et al. 2014). Comprehensive evidence-based services are essential to addressing criminogenic, health, and psychiatric needs, and ultimately altering trajectories of long-term criminal behavioral involvement and substance use and associated problems (Abram et al. 2015; D’Amico et al. 2008; Karnik et al., 2009; Epperson et al., 2011).

### Juvenile justice systems of care

Given the high rates of co-occurrence between delinquency and other behavioral problems (Huizinga et al. 2000), JJ-involved youth frequently interact with multiple service systems, such as school-based services, substance use prevention and treatment, mental health, child welfare, and health services. Across these sectors, prevalence of substance use disorders is high, especially among youth in substance use, juvenile justice, and mental health systems (Aarons et al., 2001). Cross-system linkages are essential for screening, assessing, and referring youth to needed services, either within or across systems.

In recognition of the multi-varied needs of this population, recent policy initiatives have focused on improving community-based systems of care through juvenile-justice based partnerships (Cocozza et al.^2010^; Schubert & Mulvey, 2014). Coordination of service delivery between CS and community-based BH service providers is essential to ensuring the delivery of needed services. Yet prior research has demonstrated that coordination between correctional agencies and treatment providers is often hindered by numerous organizational and programmatic barriers that impede cross-system communication, collaboration, and service delivery (Lehman et al. 2009; McCarty & Chandler, 2009). Several mechanisms for facilitating coordination across service systems have been proposed, including information exchange, cross-agency client referrals, cross-system training of staff, networking protocols, interagency councils, and service integration models (Howell et al., 2004; Trupin & Boesky, 1999).

This paper uses the Juvenile Justice Behavioral Health Services Cascade framework proposed by Belenko et al. (2017) as a framework for examining the systems of care for youth under CS and their corresponding BH service providers. This framework describes the various stages through which youth enter into the JJS system, are screened and assessed for treatment needs, referred to treatment/services, initiate treatment, and are engaged and retained in treatment/services over time. This sequential framework identifies transition points across the services cascade and service gaps that may be improved with greater cross-system coordination.

The JJS provides a wide range of opportunities for screening, assessing, treating, and referring large numbers of symptomatic youth who would otherwise have little or no access to BH care interventions (Ives et al. 2010). Moreover, the JJS is situated within the broader community system of care and provides opportunities for service integration across mental health, child protection, education, and juvenile justice agencies (Underwood & Washington, 2016). Studies have demonstrated the feasibility of implementing evidence-based screening instruments, clinical assessment tools, and therapeutic interventions as well as the effectiveness of substance use and mental health treatments, and HIV-prevention services for young people within the JJS (Grisso & Underwood, 2004; Tolou-Shams et al., 2009). Quality implementation of EBPs within JJ programs is associated with their effectiveness in reducing recidivism (Lipsey, 2009). Further, the availability of these services often fails to match the demand for care, and the rates of engagement and retention are correspondingly low (Mendel, 2011; Teplin et al., 2002; Young et al. 2007).

### Current Study

In 2013, the National Institute on Drug Abuse, National Institutes of Health, responded to the challenges of JJS-involved youth by funding a multi-component initiative known as Juvenile Justice-Translational Research on Interventions for Adolescents in the Legal System (JJ-TRIALS), https://www.drugabuse.gov/jjtrials. The purpose of the current study within the JJ-TRIALS initiative was to develop a national profile of CS agencies and their corresponding BH agencies with respect to (1) the characteristics and BH needs of the youth they serve; (2) their practices related to BH screening, assessment, and referral in the areas of substance use and HIV prevention, and (3) their practices related to provision of substance use and mental health treatment. Within each area, the study also examined the use of evidence-based practices, information exchange, and referral practices.

In a multistage sample of counties, surveys were conducted with: 1) all CS agencies, 2) the primary BH service providers affiliated with each CS, and 3) the judge with the largest docket of youth on CS. The results of the survey of judges have been previously reported (Scott et al. 2017). This article combines the data from the CS agencies and primary BH service providers to represent the “juvenile justice (JJ) system of care” in order to examine the following questions:

1. How do characteristics of youth on CS compare with youth served by their affiliated BH service providers?
2. What is the availability and range of BH services for youth on CS that are provided directly and/or by referral, and to what extent are these services either unavailable or unknown within the JJ systems of care?
3. To what extent are EBPs for JJ-involved youth used within the local systems of care, what proportion of youth receive EBPs, and what are the qualifications of staff providing EBPs?
4. How are patterns of information exchange, collaboration, and cross-system referral related to quality of BH services provided within JJ systems of care?

## Methods

### Probability sampling

Respondent selection was based on a three-stage national probability sampling process that included states, counties, and CS agencies within counties. States and counties were stratified by the number of youth aged 10 to 19 residing in them, as documented in the 2010 Current Population Survey (United States Census, 2012). In the first stage, the five largest states were selected with certainty, and the remaining 15 were selected with probabilities proportionate to the number of youth in five population strata to ensure that less-populated states were included in the study. In the second stage, within each state the largest county and any other mega-counties (with 250,000 or more youth or half or more of the state’s youth in smaller states) were selected with certainty. The remaining counties were selected with probabilities proportionate to the number of youth in those counties. In the two small sampled states where CS and BH services were organized by judicial district (vs. county), all counties in the state were selected with certainty. In stage 3, all CS agencies that served youth on CS in the 192 sampled counties were identified and surveyed regardless of the number of youth they served.

*CS Agency Recruitment and Weighting*. In states where there was direct management of CS agencies, we contacted key state-level stakeholders to identify and make a personal referral to the most appropriate CS agency contact in each county to encourage their participation in the survey. In states with decentralized systems, we identified and contacted a local leader (e.g., head of state sheriff or probation association) and asked them to do the same. In the 192 counties, 182 had one CS agency, and 10 had multiple CS agencies (9 had 2, and 1 had 3), for a total of 203 CS agencies. Surveys were completed by 195 of the 203 (96%) CS agencies.

Data were weighted based on the inverse of the inclusion probability and were adjusted for nonresponses within states. The number of agencies overall and those providing a specific service were estimated by multiplying the weighted average number of agencies per county by the number of counties (*n* = 3143). For youth characteristics, the weight was further adjusted to account for the number of youth served so that the estimate better represented youth on CS (*N* = 770,323).

### BH service provider recruitment and matching

Each of the selected CS agencies was asked to identify the primary BH service providers of substance use and mental health treatment they used based on the number of youth under CS from their sampled county. This could be one or two providers and/or an internal unit of the CS agency. A total of 283 BH providers were identified, and of these, 271 surveys (96%) were completed and returned.

BH provider data was merged with the CS agency data at the CS agency level in the following way. Within counties, BH providers were matched with the CS agency that identified it as the main service providers for that agency. When there were multiple CS agencies per county (e.g., county and state-based CS), the identified BH providers were matched with their corresponding agencies. The same method was used when judicial districts were used within a state for CS agencies, instead of counties. If a single BH provider was identified by more than one CS agency within a county, that record was duplicated and matched to each CS agency. The average unweighted number of BH providers to CS agency is 1.4 and ranged from 0 to 2 (0 for 10 CS agencies). The 0 s included 6 CS agencies that were themselves the primary (direct) BH service provider. For these cases, their responses to survey items were also used to represent the BH service provider.

In the cases (*n* = 86) where there were separate service providers of substance use and mental health treatment, their data was aggregated into a new BH service provider record. For dichotomous items (0/1 for no/yes), the max across BH providers was used to create the matching BH provider variable for that CS agency’s record. For continuous items, such as the percentage of youth served, the average across BH agencies was used to create the new BH matched version of the variable. After aggregating the BH provider data to their corresponding CS agency, the final data set has 195 JJ system-of-care records for the main analysis, which are then weighted to estimate the 3202 JJ systems of care in the U.S.

The total number of BH service providers overall was estimated based on the weighted average number of BH service providers per county times the number of counties (*n =* 3143). The number of BH providers providing each specific service were estimated by multiplying the weighted average number of providers of each service times the number of BH service providers (*n* = 4252). For youth characteristics, the service provider weight was multiplied by the number of youth served to represent the estimated number (*n* = 548,613) of youth on CS seen by this primary BH service provider. Overall, data are weighted to reflect the national population estimate of the 4252 primary BH service providers and 3202 JJS CS agencies in the 3143 counties in the United States, and have been adjusted for survey non-response at the state level.

### Survey domains and development

The survey items were drawn largely from validated tools as well as studies, guidebooks, and compendiums that contained scientifically grounded information on the assessment of juvenile offenders. See Additional file 1 for a complete list of these sources.

A JJ-TRIALS survey advisory board of researchers, national association directors, juvenile justice researchers, and representatives from each of the JJ-TRIALS research centers met several times to review the study’s survey, identify problem items, clarify definitions of terms, prioritize items for inclusion, and suggest overall revisions to item wording and sequencing. The penultimate instrument was sent to several CS agencies for pilot testing and further revision. The first dozen completed surveys were also reviewed very closely. Unclear answers to questions were clarified with respondents’ input. Sources of confusion that stemmed from unclear words or instructions as well as ambiguous definitions of terms were removed from the instrument.

Lists of EBPs were based on the peer-reviewed programs enumerated in the federal National Registry of Evidence-Based Practices and Programs, and in Crime Solutions that were rated as having promising or strong evidence. Any practices rated as ineffective or harmful were included in the list of practices, but not in the measure of evidenced-based practice used by the agency. Other EBPs were identified and approved by members of the JJ-TRIALS cooperative. In each list of EBPs, respondents could also identify that they used locally developed measures (not counted as EBPs) as well as any “other evidence-based practices that they used for ___.” These responses were reviewed and coded by two investigators into “other EBP” or other categories of responses (“non-behavioral health EBP,” “not an EBP,” or “unknown”). The inter-rater rate of agreement was 70% with a Kappa of .62. The raters then reviewed and resolved any discrepancies.

The JJS CS and BH surveys each contained 13 sections that included questions on data availability; agency characteristics; youth characteristics; BH (substance use, HIV, and mental health) screening, clinical assessment and referral; substance use and HIV/STI-risk prevention; substance use and mental health treatment; and interagency collaborative activities, family engagement and technical assistance needs. The questions also focused on whether services were provided directly or through referrals; the names and utilization of EB tools, protocols, and other practices; and staff educational levels.

### Item wording and formatting

The survey included a variety of question types, including both “choose one” and “choose all that apply” from lists. These questions were always followed by an “other” response, allowing participants to write in more detailed or individualized information. Still other questions asked participants to respond numerically (e.g., number of staff, number of youth served) or to rate items using a Likert scale. Agency representatives were asked about the availability in their county of each service listed in Table 2. Specifically, they were asked to check all the following options that applied to each of the services:

- They don’t know where youth can access the service in the county.
- The service is not available in the county.
- Their agency provides the service directly to youth, or
- The service is provided by an external agency.

If they provided the service directly, they were asked how many youth on CS received the service and what minimum level of staff education was required of the person administering the service. In describing their use of EBPs, respondents reviewed a list of EBPs for each type of service (prevention, substance use treatment, mental health treatment) and were asked to identify the practices they have implemented. Respondents were also asked to list any implemented practices that they developed on their own. A detailed list of the practices that were queried for each service area can be obtained from the first author.

### JJ system of care composite measures

The following composite indices of cross-system interactions and collaboration were created.

1. The *CS to BH Referral Assertiveness* items were collapsed across 14 referral activities CS agencies reported performing to facilitate referrals for substance use and/or mental health problems (1 if done with either or both types of providers, else 0). The scale score was the percentage of the 14 items endorsed, and demonstrated good internal consistency with a Cronbach’s alpha of 0.79.
2. The *CS Information Received from BH* measure was the average of youth per CS agency the agency received information about from BH service providers. First, the average was calculated across 8 areas of information the CS agency received from substance use and mental health treatment providers. These include admission and discharge dates, discharge status and summary reports, monthly progress reports, dates of missed appointments, results of urine or other biological tests, and the amount of services received. The alpha across these eight items was high, at 0.98.
3. The *CS Quality of Direct BH Services* measure is based on the count of: 1) whether a CS agency directly provides a service, 2) if that service is evidence-based, 3) is the evidence-based service provided to 50% or more of the youth served, and 4) is there an educational requirement of at least a bachelor’s or nursing degree for those implementing the service. This count goes across all services: screening, clinical assessment, substance use prevention, HIV risk behavior prevention, substance use treatment, and mental health treatment. Altogether, there were 24 items with an alpha of 0.88.
4. The *BH Information Sent to CS* measure is an average per agency across 8 items that ascertain the percentage of youth for which the CS agency received information from the BH agencies. Again, there was high internal consistency, with an alpha of 0.96.
5. The *Quality of BH Direct BH Services* is calculated in the same manner as no. 3, with regard to BH services provided by the BH provider. For these 24 items, the alpha was 0.75.
6. The *CS to BH Collaboration Scale* is based on 11 activities the CS agency reported doing with external BH agencies to help facilitate services to youth on CS. Items were collapsed across both substance use and mental health items by taking the max (1 if done with either or both types of providers, else 0). The scale score was the percentage of the 11 activities endorsed and had an alpha of 0.79.
7. The *BH to CS Collaboration Scale* is calculated in the same manner as no. 6 above with regard to 11 activities, except from the perspective of the BH provider with regard to their collaborative activities with CS agencies. For these 11 items, the alpha was 0.81.

The two collaboration measures above (nos. 6 and 7) were correlated (r = 0.51) but measured different functions. They were dichotomized into low and high groups based on median splits. These two dichotomies were then used to create four separate groups based on CS low/high on collaboration and the BH low/high on collaboration. These four groups include: (1) CS Low/BH Low (*n* = 1229), (2) CS High/CS Low (*n* = 666), (3) CS Low/BH High (*n* = 536), and (4) CS High/BH High (*N* = 771). Differences across these groups on each scale were examined by F test, effect size using the f-index [small = 0.10, moderate = 0.25, large = 0.40] (Cronbach, 1960), and percentage of variance explained using eta-square.

### Survey administration

All of the CS agencies in the sampled counties were contacted to participate in the survey. The breadth of the CS survey often required input from diverse agency staff with access to different information. To identify the most appropriate staff member for completing the survey, each state was assigned a survey coach who contacted each agency’s key stakeholder and provided an overview of the survey components. During this phone conference, the survey coach and stakeholder identified the best respondent for answering each set of questions and the best available data sources for completing particular survey items. A similar process was used for the BH agencies, in which the survey coach contacted the CS provider’s key BH stakeholder, provided an overview of the survey components, and identified the best respondent for answering each set of questions and the best available data sources for completing particular survey items.

A survey coach then delivered a PowerPoint presentation to the potential respondents which described the survey’s goals and data-collection process. In addition, the survey coaches mailed the surveys and made survey-orientation calls to all respondents who had agreed to participate in the study. At the end of these calls, the coaches and respondents agreed on a completion date for the survey. Coaches also contacted respondents each week to answer any questions and to obtain updates on the progress of the surveys.

Upon receipt of completed surveys, survey coaches reviewed the surveys, and respondents were queried about missing data, inconsistent responses, or notes from participants with questions about the items. Surveys were rekeyed to reach at least 99% intra-survey agreement, and 1 in 6 was rekeyed. The rekey agreement rate was 98.9% for the CS agency surveys and 99.0% for the BH survey provider surveys. The surveys were reviewed a final time by the lead analyst to remove or resolve any remaining inconsistencies. The Cronbach’s Alpha for 16 of the 17 survey sections that consisted of correlated items was .7 or higher (5 at .9+, 8 at .8 to .89, and 3 at .7 to .79), with one section (Family Systems Engagement) attaining an Alpha = .67.

### Analytic methods

Data were analyzed with IBM’s Statistical Package for Social Sciences (SPSS) Version 25 frequencies and descriptive procedures, using the national weights described above for agencies (inverse of state selection, county selection, and adjustment for non-response) and youth (agency weight times the estimated number of youth served). Descriptive data presented in Tables 1, 2 and 3 show prevalence and estimated sample size, which is based on the prevalence multiplied by the number of estimated agencies. Differences between prevalence estimates were evaluated using odds ratios (OR). The denominator for a sample subset is presented in the table notes. The figures are all weighted as well. SPSS multivariate Generalized Linear Model (GLM) was used to test differences on each scale based on the four collaboration groups formed by crossing the two median split variables described above, specifically: (1) CS Low/BH Low (*n* = 1229), (2) CS High/CS Low (*n* = 666), (3) CS Low/BH High (*n* = 536), and (4) CS High/BH High (*N* = 771). Results of these multivariate analyses, presented in Table 4, report the means of each scale by the four groups, the F- statistic, *p*-value, and eta-square. Effect size is evaluated using the f-index (which we have interpreted as 0.10 = small, 0.25 = moderate, and 0.40 = large) reported in Table 4. To avoid inflating the power of this analysis, the weighted n’s were reduced to maintain the weighted proportions, but add up to the raw n of observations.

**Table 1:** Data Availability and Rates of Youth Characteristics by Type of Provider\a

	Community Supervision Providers		Behavioral Health Providers	
Youth Characteristic	Data Available	Prevalence	Data Available	Prevalence
Demographics				
Male	84%	73%	80%	67%
Female	84%	27%	80%	33%
Under age 14	76%	12%	77%	18%
Age 14–15	79%	36%	77%	27%
Age 16–17	79%	44%	77%	39%
18 or older	81%	8%	78%	12%
White/Caucasian	81%	53%	77%	50%
Black/African American	80%	26%	77%	19%
Other or Mixed Race/Unknown	70%	6%	75%	16%
Hispanic/Latino Ethnicity	79%	18%	77%	21%
Substance Use Problems				
Any substance use problems including alcohol	46%	51%	71%	66%
Marijuana use problems	35%	49%	65%	59%
Alcohol use problems	40%	25%	66%	41%
Prescription drug misuse	25%	19%	50%	16%
Other drug use problems (e.g., amphetamine/methamphetamine, cocaine/crack, opioids/heroin, hallucinogens/K2/Salts)	35%	18%	53%	18%
Tobacco use problems	24%	32%	48%	51%
Mental Health Problems				
Serious Family Problems (e.g., substance use, serious mental illness, domestic violence, incapacitating chronic illness)	31%	60%	58%	67%
Internalizing Mental Disorders (e.g., Mood, Depression, Anxiety, Trauma, Posttraumatic Stress Disorder, Psychosis)	30%	35%	70%	57%
Externalizing Mental Disorders (e.g., Attention deficit, hyperactivity, conduct, pathological gambling, or other impulse control disorder)	26%	35%	67%	52%
Learning Disabilities or Other Cognitive Impairment	25%	28%	47%	28%
Suicide Risk (e.g., self-mutilation, thoughts, plans, means, attempts)	31%	13%	56%	22%
Other Behavioral Health Problems				
Risky sexual activity (e.g., unprotected sex, sex under the influence, multiple sex partners, sex trading, sex with high risk partners)	17%	51%	39%	44%
Physical, Sexual or Emotional Victimization	29%	39%	57%	44%
Violence towards other	32%	25%	46%	36%
Physical Health Problems	21%	11%	56%	12%
Needle related risk activity (e.g., use, old or unclean needles, sharing, sharing with risky partners)	14%	2%	40%	3%

\a Data are weighted to reflect the estimated national population estimate of the 3202 CS agencies and 4252 BH service providers and in the U.S. and have been adjusted for survey non-response at the state level

**Table 2:** Availability and Mode of Provision of Behavioral Health Services for Juvenile Offenders\a

Service	CS Directly Provides	BH Directly Provides	External Provider/Other	Not Available/ Don’t Know
Prevention				
Substance Use Prevention	17%	55%	39%	1%
HIV/AIDS testing	0%	4%	94%	2%
Tuberculosis, Hepatitis B, Hepatitis C testing	1%	4%	93%	3%
Sexually transmitted infection (STI) testing	0%	3%	94%	3%
STI prevention, education and counseling	2%	23%	73%	3%
Hepatitis prevention, education, and counseling	0%	22%	75%	4%
HIV prevention, education and counseling	2%	25%	64%	9%
Substance Use Treatment				
Outpatient	9%	93%	6%	1%
Co-occurring substance and mental health treatment	5%	80%	17%	3%
Continuing or aftercare	4%	68%	25%	10%
Intensive outpatient	1%	39%	48%	15%
Other recovery support	1%	25%	55%	21%
Residential treatment	1%	10%	65%	25%
Medication assisted treatment	0%	7%	62%	31%
Detoxification	0%	4%	57%	39%
Mental Health Treatment				
Individual counseling	9%	91%	10%	0%
Family counseling	11%	87%	14%	0%
Group counseling	4%	76%	24%	2%
Medication assisted treatment	1%	74%	29%	0%
Residential treatment	1%	7%	76%	18%
Day program	< 1%	18%	64%	19%

\a Data are weighted to reflect the estimated national population estimate of the 3202 CS agencies and 4252 BH service providers and in the U.S. and have been adjusted for survey non-response at the state level

**Table 3:** Service Provision and Use of Evidence-Based Practices by Type of Provider\a

	National Estimate of Community Supervision Agencies Providing		Row % of Agencies Providing		
	No. of CS Agencies	% of all Providers	Used EBP	Used EBP on 50% or more of youth	Provided by clinical staff with Bachelor’s degree or above
Screening	2034	64%	86%	78%	29%
Clinical Assessment	772	24%	98%	49%	84%
Substance Use Prevention	544	17%	48%	15%	92%
HIV Testing and Prevention	96	3%	< 1%	< 1%	86%
Substance Use Treatment	365	11%	95%	33%	90%
Mental Health Treatment	431	13%	86%	18%	100%
Both Substance Use and Mental Health Treatment	253	8%	86%	37%	100%
	National Estimate of Behavioral Health Agencies Providing		Row % of Agencies Providing		
	No. of BH Agencies	% of all Providers	Used EBP	Used EBP on 50%or more of youth	Provided by clinical staff with Bachelor’s degree or above
Screening	2503	78%	95%	90%	96%
Clinical Assessment	3107	97%	86%	85%	98%
Substance Use Prevention	1790	56%	39%	6%	80%
HIV Testing and Prevention	853	27%	18%	7%	96%
Substance Use Treatment	3096	97%	90%	90%	97%
Mental Health Treatment	2966	93%	98%	73%	100%
Both Substance Use and Mental Health Treatment	2622	82%	98%	92%	100%

\a Data are weighted to reflect the estimated national population estimate of the 3202 CS agencies and 4252 BH service providers and in the U.S. and have been adjusted for survey non-response at the state level

**Table 4:** Mean of Collaboration Measures by CS and BH Collaboration Groups

	Collaboration groups based on low (0–0.36) and high (0.37+)								
	Total	CS Low/ BH Low	CS High/ BH Low	CS Low/ BH High	CS High/ BH High	*F*	*p*	f-index	eta-sq.
Unweighted n	185	33	40	28	84				
Weighted n	3202	1229	666	536	771				
Weighted %	100%	38%	21%	17%	24%				
Weight Adjusted n	185	71	38	31	45				
CS to BH Referral assertiveness (% of 14 items)	63%	53%	76%	60%	72%	19.08	**0.000**	**0.57**	**0.25**
CS Information received from BH (% of 8 items)	49%	53%	48%	23%	65%	7.71	**0.000**	**0.36**	**0.12**
CS Quality of direct BH services (% of 24 items)	11%	9%	13%	11%	14%	1.40	0.246	**0.15**	**0.02**
BH Information sent to CS (% of 8 items)	52%	45%	54%	52%	64%	2.33	0.076	**0.20**	**0.04**
BH Quality of direct BH services (% of 24 items)	60%	59%	58%	64%	63%	1.01	0.389	**0.13**	**0.02**
Average across above	47%	44%	50%	42%	55%				

The 4 groups explain 39% of the variance in the joint distribution of these measures of collaboration (1-Wilk’s lambda)

Effect size f-index: small = 0.10, moderate = 0.25 and large = 0.40

CS = community supervision; BH = behavioral health

Bold indicates *p* < .001, f-index > 0.10, eta-sq > .01

## Results

### CS agency characteristics

Of the 3202 CS agencies in the United States, 27% operate under a state judicial branch; 39% under a state executive branch; 25% under a county, municipal, or local judicial branch; 6% under a municipal or local executive branch; and 3% under other authorities. Approximately 32% had a specialty court, with the most common being a juvenile drug treatment court (12%), family drug treatment court (6%), peer court (5%), teen court (4%), or mental health court (2%). In terms of the legal minimum age of youth that CS agencies could supervise, 42% specified no lower age limit, 22% specified nine years or younger, 20% specified 10 or 11 years, and 17% specified 12 or 13 years. Regarding the legal maximum age of youth that CS agencies could supervise, 5% specified 14 to 16 years, 38% specified 17 years, 25% specified 18 to 20 years, 24% specified 21 years or older, and 8% specified no upper age limit.

#### CS employees and their educational level

The average CS agency employed a staff of 10 full-time equivalents (FTEs) working with an average of 240 youth on CS. Approximately 55% of all youth were seen in a subset of 18 CS agencies that were larger, averaging 200 FTEs working with an average of 4406 youth on CS. Almost all (92%) of the CS agencies employed non-clinical staff, and almost a fourth (22%) employed master’s-level clinicians in order to serve youth on CS. Other staff positions included bachelor’s-level clinicians (19%) and registered nurses (6%).

### BH service provider characteristics

#### Accreditation and funding sources

Nationally there were 4252 primary BH service providers for youth on CS. Of these, 65% operated under the auspices of private non-profit organizations, 20% under private for-profit organizations, 9% under county or other local government agencies, and 1% under state government agencies. Slightly more than half of the service providers (52%) reported being accredited by a state mental health department, nearly half (47%) by a state substance use department, and 41% by the Commission on Accreditation of Rehabilitation Facilities (CARF). Other accreditation bodies included the State Department of Health (27%), the Joint Commission (11%), the Council on Accreditation (3%), and a hospital licensing authority (1%). Service providers received funding from a variety of sources including: self-paying clients (75%); private insurance (74%); state (69%), county (48%), and federal governments (39%); private donations (24%); and local (municipal) government entities (8%). Approximately 2% were a BH service unit within a CS agency.

#### BH employees and their educational level

The average BH service provider employed a staff of 15 FTEs working with an average of 174 youth on CS. Half of all youth were seen in a subset of 33 (0.8%) BH providers that were larger, averaging 98 FTEs working with an average of 2664 youth on CS. Almost all (93%) of the BH providers employed master’s-level clinicians, and three-fourths (76%) employed non-clinical staff in order to serve youth on CS. Other staff positions included psychiatrists (54%), bachelor’s-level clinicians (54%), registered nurses (45%), doctoral-level clinicians (36%), non-degreed clinical staff (31%), physician’s assistants (19%), and physicians (5%).

### Characteristics and needs of youth served within JJ systems of care

Table 1 presents the percentages of CS agencies and the corresponding BH providers with data available on and prevalence of youth demographic characteristics and their substance use, mental health, and other BH problems. Estimates were adjusted for non-response/data unavailability and the number of youth served by each agency; thus, the estimates represent the 770,323 youth on CS in the United States. It is important to note that the JJ-involved youth referred from CS to BH providers are a subset of all youth who are under CS. As such, we expected the characteristics of youth served to be generally similar across the two systems, although skewed toward higher severity of BH problems among youth who are referred into BH programs. Because the youth served in the CS and BH systems are not independent, statistical tests of differences are not conducted; however, we note below variables on which there was a deviation of approximately 10% between CS and BH prevalence estimates or ORs of ≥ 2.0.

#### Demographic characteristics

Between 70% and 84% of the CS agencies and 75% - 80% of the BH providers had data available for youth demographic characteristics. Most youth were male (73%), Caucasian/White (53%), and between the ages of 14 and 17 (80%). Based on the 2010 US Current Population Survey, the proportions of youth identified as Black/African American (26%) or other/mixed race (6%) were higher than those identified in the general population. The proportion of Hispanic/Latino (18%) youth was similar to the proportion in the general population. Relative to all youth seen by CS agencies, those seen by BH service providers were less likely to be aged 14–15 (36% vs. 27%, OR = 0.65); youth in the BH providers were also more likely to be of other or unknown/mixed race relative to those in CS programs (16% vs. 6%, OR = 2.94).

#### Substance use problems

Between 24% and 46% of the CS agencies and 48% and 71% of the BH providers had data available on substance use problems. Relative to all youth seen by CS agencies, estimates of prevalence of substance use problems for youth seen by BH service providers were consistently higher, including for any substance use problem (51% vs. 66%, OR = 1.90), marijuana problems (49% vs. 59%, OR = 1.47), alcohol problems (25% vs. 41%, OR = 2.06), and tobacco problems (32% vs. 51%, OR = 2.25). Estimates of misuse of prescription drugs (19%, 16%) and other substance use (18%) were similar.

#### Mental health

Between 25% and 31% of the CS agencies and 47% and 70% of the BH providers had data available on youth mental health needs. Relative to all youth seen in CS, the subset of youth seen by BH service providers had higher levels of mental health problems, including for internalizing disorders (57% vs. 35%, OR = 2.42), externalizing disorders (52% vs. 35%, OR = 2.05), and suicide risk (22% vs. 13%, OR = 1.88). Estimates for serious family problems (60%, 67%) and for learning disabilities and other cognitive impairments (28%) were similar.

#### Other behavioral health problems

Between 14% and 32% of the CS agencies and 39% and 57% of the BH agencies had data available on other BH problems. Relative to all youth seen in CS, the subset of youth seen by BH service providers reported significantly higher rates of violence toward others (25% vs. 36%, OR = 1.72). Estimates of risky sexual behaviors (44%, 51%), physical, sexual, or emotional victimization (39%, 44%), physical health problems (11, 12%), and or needle-risk behaviors (2%, 3%) were similar across CS and BH providers.

### Patterns of service delivery and availability within JJ systems of care

**Fig. 1:**
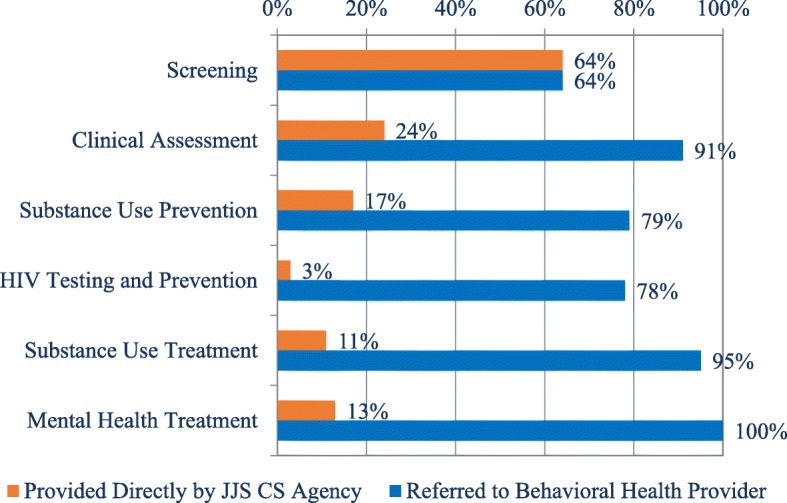
Services provided to youth in juvenile justice system directly or through referral (weighted percentage of 3202 JJ community supervision agencies). Orange bar = Provided Directly by a JJS CS Agency; blue bar = Referred to Behavioral Health Provider

Figure 1 shows the percentage of CS programs that directly provide six types of BH services as well as referrals for youth to external providers for these services. Although nearly two thirds of CS agencies (64%) directly screened youth for various BH problems, an overlapping 64% referred youth to other agencies for BH screening as well. In contrast, few of the CS agencies directly provided additional screening, clinical assessment, substance use prevention, HIV prevention and testing, substance use treatment, and mental health treatment.

The next set of analyses examines the service delivery pattern complexity of the JJ systems of care. The first 3 columns of Table 2 show the percentages of times each service was provided directly by the CS agency, the BH service provider, or another agency (e.g., public health department). The last column is the combined percent of times when both the CS agency and/or BH service provider indicated that the service was not available in the county or that they did not know about its availability.

#### Prevention services

Few CS or BH programs directly provided prevention services, with the exception of substance use prevention, which was provided by 55% of the BH service providers and 17% of the CS agencies. Youth were referred to other providers for substance use prevention (i.e., no direct provision in either CS or affiliated BH programs) among 39% of the respondents.

Few CS agencies directly provided testing, prevention, education, or counseling for HIV, other STIs, or infectious diseases. About one-quarter (23%) of the BH service providers provided infectious disease prevention, education, and counseling (but not testing). A majority of the CS and affiliated BH providers referred youth to external providers for these services; less than 10% reported that these services were either unavailable in the county or did not know where the services could be found.

#### Substance use treatment

Overall, 11% of CS agencies and 97% of the affiliated BH programs directly provided some form of substance use treatment. The most common form of substance use treatment by BH providers was outpatient (93% of BH). A large proportion of the BH providers (80%) provided treatment for co-occurring substance use and mental health disorders, whereas 17% of the affiliated CS and BH providers referred youth to other providers for co-occurring disorder treatment. Close to two fifths of the BH providers directly provided intensive outpatient treatment, and close to half (48%) of the affiliated CS and BH providers did so through external referral. Few BH providers (7%) offered medication-assisted treatment, although it was often provided through external referrals (62%). Residential treatment and other recovery support services were most frequently provided through external referrals (65% and 55%, respectively). A substantial portion of the affiliated providers indicated that the following services were either non-existent in the county or unknown to them: recovery support services (21%), residential treatment (25%), medication-assisted treatment (31%), and detoxification (39%).

#### Mental health treatment

Overall, 13% of CS agencies and 93% of BH agencies directly provided some form of mental health treatment. Most common among CS agencies was provision of individual (9%) and family counseling (11%); less than 5% provided other forms of mental health treatment. Instead, a majority of the affiliated BH programs directly provided counseling services, including individual (91%), family (87%), and group (76%), as well as medication (74%); youth were less often referred to external providers for counseling or medication services. Fewer BH programs provided day treatment programs (18%) or residential treatment (7%), while a majority of the affiliated CS and BH providers indicated these more intensive mental health services were provided through external referral (64%, 76%, respectively). However, approximately one fifth (18%, 19%) indicated that these services were either not available in the county or they did not know if they were available.

#### Use of EBPs

Data on the provision of EBPs by CS providers are shown in the top half of Table 3 and for BH providers in the bottom half. The first two columns show the weighted subset of agencies providing each type of BH service. The next three columns show the weighted percentages of the subset that reported using one or more EBPs, implementing these EBPs with at least one half of the youth in their programs, and delivery of EBPs by clinical staff who held bachelor’s degrees, registered nurse qualifications, or higher degrees.

#### Community supervision agencies

Although direct provision of services is generally low among CS agencies (with the exception of screening), among those CS agencies that reported providing screening, assessment, and BH treatment, most (> 85%) used EBPs. Less than one-third (32%) of CS agencies that provided substance use prevention services used EBPs, and virtually none used EBPs for HIV testing and prevention. Although a majority of CS agencies provided screening, and most of these (78%) used EPPs with at least half of the youth, few (29%) reported using clinical staff with bachelor’s, nursing, or higher-level degrees. In contrast, less than one-quarter of the CS agencies provided in-depth assessment, prevention, or treatment services; among those that provided these services, most used EBPs, although less than half did so with 50% or more of the youth in their programs. Most CS agencies that used EBPs reported employing staff with higher levels of education (ranging from 84% for clinical assessment to 100% for mental health treatment and both substance use and mental health treatment).

#### Behavioral health agencies

Use of EBPs was more comprehensive in the affiliated BH agencies. With the exception of prevention services, nearly all BH agencies that provided screening, assessment, and treatment services used EBPs with at least half the youth in their programs. Similarly, agencies that reported using EBPs for screening assessment, prevention, and treatment reported these services were delivered almost always by clinical staff with higher levels of education.

### Interactions across CS agencies and BH service providers

#### Cross-system collaborative activities

**Fig. 2:**
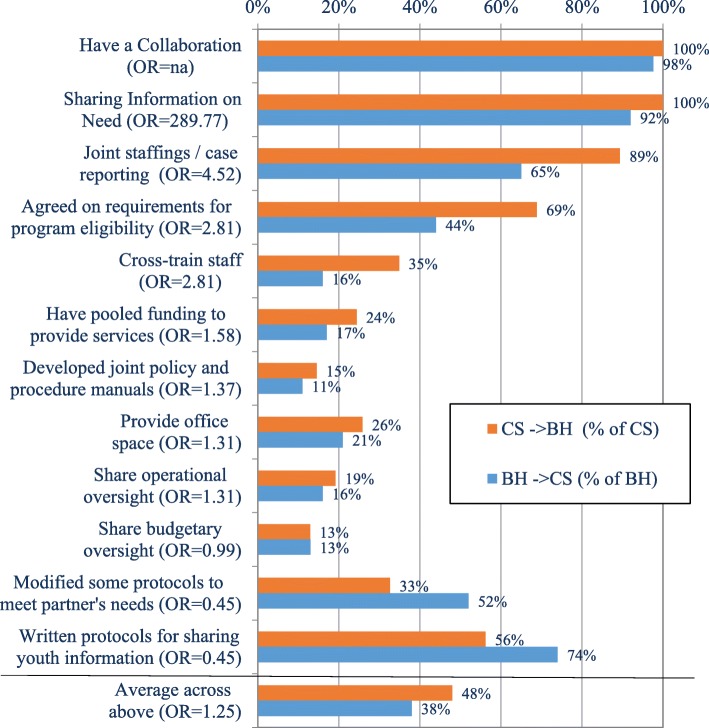
Collaborative Activities to/from CS and BH Agencies. Orange bar = CS - > BH (% of CS); blue bar = BH - > CS (% of BH)

Collaborative activities between CS agencies and BH service providers within JJ systems of care are displayed in Fig. 2. Nearly all CS agencies and BH providers reported that they did indeed have cross-system collaborations; most commonly they reported sharing information on client needs (100% and 92%, respectively). A majority of both CS agencies and BH service providers also reported having joint staffings/case reporting (89% and 65%, respectively) and written protocols for sharing information on clients (56% and 74%, respectively). Although 69% of CS agencies stated there was agreement on requirements for program eligibility, only 44% of the BH service providers concurred with this statement. Fewer than half of the affiliated CS and BH providers reported that they cross-train staff, modify some protocols to meet service partner needs, provide office space, have pooled funding to provide services, share operational oversight, have developed joint policy and procedure manuals, or share budgetary oversight. Overall, the CS agencies and BH service providers endorsed an average of 44% and 38%, respectively, of the collaborative activities.

#### Activities CS agencies routinely complete to facilitate referrals

**Fig. 3:**
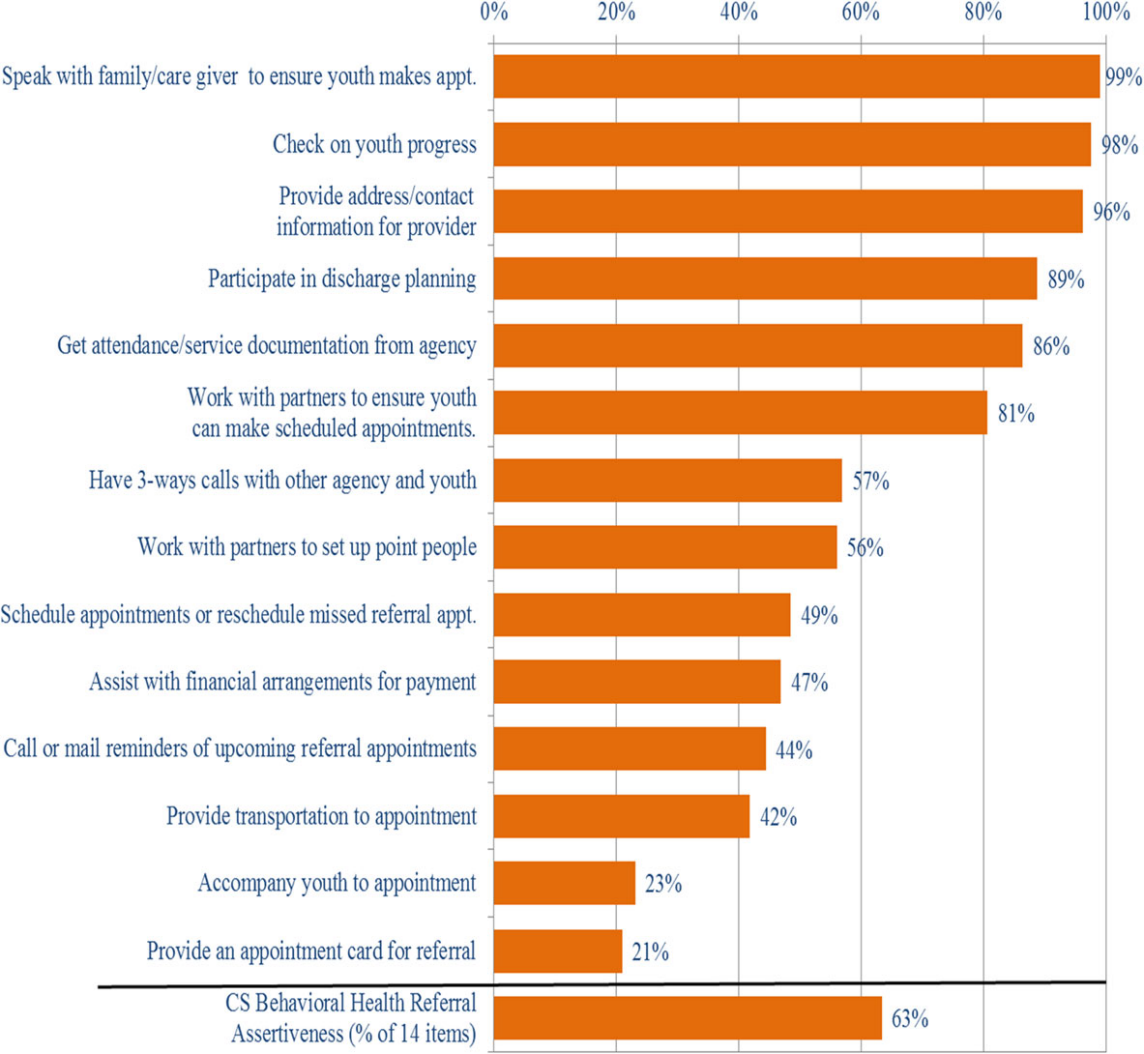
Assertiveness of Referral Activities from CS to BH Agencies

Among CS agencies, the most common referral practices reported to facilitate linkages with BH providers (regarding either substance use or mental health referrals) were speaking with a family member or caregiver to ensure that youth attended their appointments (99%), checking on youth progress (98%), and providing the caregivers with contact information for the service providers (96%) (See Fig. 3). A large majority of CS agencies also reported that they participate in discharge planning (89%), obtain attendance/service document from the partner agency (86%), work with service partners to ensure that youth were able to attend scheduled appointments (81%), have 3-way calls with the other agency and the youth (57%), and work with service partners to set up point people to coordinate care for the youth being referred (56%). Fewer than half of the CS agencies reported that they schedule appointments or reschedule missed appointments (49%), assist with financial arrangements for payment, provide appointment reminders (47%), arrange for transportation to an appointment (42%), accompany the youth to appointments (23%), or provide the youth with appointment cards (21%). Overall, the CS programs endorsed an average of 63% of the 14 items that facilitate referral.

#### Services-related information transmitted from BH service providers Back to CS agencies

**Fig. 4:**
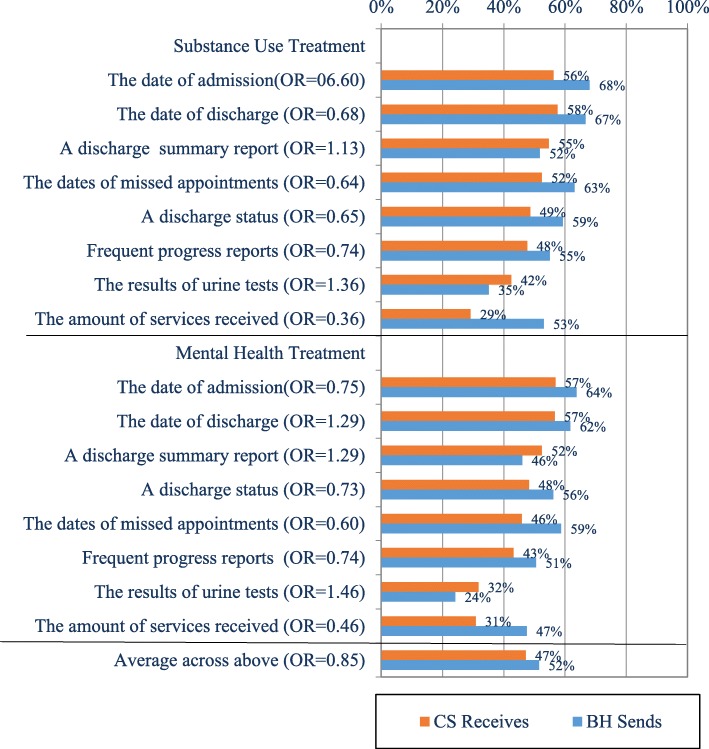
Services-Related Information Transmitted from BH and Received by CS Agencies. Orange bar = CS Receives; blue bar = BH Sends

The percentage of youth for whom information is transmitted from BH to CS, and conversely, is received by CS from BH is shown in Fig. 4. Information is most commonly transmitted from BH to CS programs regarding dates of admission and discharge, a discharge summary report, monthly or more frequent progress reports, dates of missed appointments, and discharge status. In contrast, information on urine or other biological tests and the amount of services received by the youth is less often sent from BH service providers to the CS agencies. On average, BH programs provide information to CS agencies on about half (52%) of the youth they serve, and similarly, CS agencies report receiving information from BH providers on about half (47%) of the youth they serve.

### Comparisons across CS and BH collaboration groups

In order to evaluate the JJ systems of care, composite indices were created on which affiliated CS agencies and BH service providers were classified separately as “high” or “low” on each of the following scales: (1) *CS Referral Assertiveness*, (2) *CS Information Received from B*H, (3) *CS Quality of Direct BH Services* (e.g., use of EBP), (4) *BH Information Sent to CS*, and (5) *BH Quality of Direct BH Services*. Table 4 shows the four high/low classification groups (in columns) by each of these five measures (in rows) with the mean % of items endorsed in the cells. Differences across the four CS-BH collaboration groups formed based on a median split of the average percentage of items endorsed on each collaboration scale were examined using generalized linear model (GLM) analysis and model fit was evaluated with, probability of alpha, Cohen’s f index, and eta square.

The four groups explained 39% of the variance in the joint distribution of these measures of collaboration. In particular, there were significant effects on *CS Referral Assertiveness* (Cohen’s f = 0.57) and *CS Information Received* (f = 0.36), and trends (Cohen’s effect size > 0.10) for the other 3 measures. The percent of items endorsed was in general highest for the CS high/ BH high – followed by CS high/BH low (which was strong on *CS Referral Assertiveness, CS Quality of Direct BH Service,* and *BH Information Sent to CS*), CS low/BH low (which was still strong on *CS Information Received from BH*), and CS low/ BH high (which was only above average on the *BH Quality of Direct BH Services*).

## Discussion

This study examined BH-need profiles of JJ-involved youth on CS, services provided and referred, use of EBPs, and collaborative activities across a representative national sample of CS agencies and their affiliated BH service providers within local JJ systems of care. The study utilized a parallel instrument across providers in the two service systems and analyzed findings by anchoring the BH providers to the corresponding CS agency within local systems (e.g., mainly counties) in order to derive weighted national estimates of system-level CS and BH providers. Prior studies have documented gaps in mental health services available for youth in juvenile detention centers, but have not examined cross-system referral and service provision (Osterlind et al. 2007; Pajer et al. 2007). The current study advances our understanding of the degree to which the services cascade is implemented across CS and BH providers, and areas that remain to be addressed in order to strengthen collaboration and improve service delivery across these systems.

As would be expected, the characteristics of youth served within the CS programs and their corresponding BH providers are similar demographically, although BH providers serve a more severe sub-group of JJ-involved youth, with a higher reported prevalence of BH problems. It is important to note that data availability was far from comprehensive in both systems, although a greater proportion of BH providers reported data for all types of BH problems. The greater availability of prevalence data in BH programs may reflect the fact that most CS programs screened and referred youth to BH providers for clinical assessments, a practice that is congruent with the survey findings that there is a greater availability of more highly trained clinical staff within BH programs that use EBP clinical assessments. Thus, although still not universal, the survey findings showed that BH screening and assessment is well established through linkages between CS and BH providers in most jurisdictions.

Overall, substance use prevention services are directly provided by few CS programs and by only slightly over half of BH programs. Further, among those programs that provided prevention services, only a minority utilized EBPs, and rarely were they offered to a majority of the youth served. This contrasts with a prior analysis of JJ-TRIALS survey data showing that most staff in JJ programs rated substance use prevention services as very important and congruent with their agency’s mission (Sales et al., 2018). Clearly, expansion of substance use prevention services is warranted in both CS and BH programs, given the opportunities for intervening with this high-risk population. In addition, most CS and BH agencies relied upon referral to external providers for HIV and other infectious diseases prevention services, and use of EBPs for screening and prevention of HIV was rare. Yet, compared with their non-criminally involved counterparts, JJ-involved youth are at greater risk of HIV/STIs due to their risky sexual and drug-use behaviors (Teplin et al. 2003). Moreover, African American and Latino JJ-involved youth are disproportionally represented among the newly diagnosed with HIV. Given this need, it is critical that existing EBPs for HIV/STI prevention are adapted for JJ-involved youth and more widely implemented in these settings.

Improving the delivery of evidence-based BH services is acknowledged as a critical component within a rehabilitative orientation to juvenile justice (Thomas et al. 2005; McCord et al. 2001). The JJ-TRIALS surveys demonstrated that there were inverse relationships between the CS and BH systems, indicating that cross-system treatment referral for delivery of BH services has been broadly adopted across these service systems. A majority of CS programs reported use of EBPs to screen youth for BH problems. Direct provision of BH treatment was rare within CS programs; instead, a majority of CS programs referred youth to BH providers for substance use and/or mental health treatment. This approach is consistent with current policy initiatives that emphasize the broader system-of-care for delivery of BH services to JJ-involved youth, given the limited capacity of the JJS to respond to the complex BH needs of this population (Odgers et al. 2005).

Outpatient substance use treatment was most commonly provided in both systems, with less access to more intensive forms of substance use treatment (e.g., intensive outpatient, residential, medication-assisted treatment). Similarly, individual and family counseling was widely available within BH programs, although more intensive forms of mental health treatment were either unavailable or unknown among one-fifth of the combined providers. The sparse availability of more intensive BH treatment suggests high risk for relapse and recidivism for youth with severe substance use and mental health problems, who are most likely to have persistent BH problems that lead to repeated cycles of criminal justice system contact (Ramchand et al., 2009; Schubert et al. 2011).

With regard to aftercare services, few JJ systems of care provided recovery support services to youth, with most relying upon external referrals for this service. One-fifth stated that recovery support for JJ-involved youth in their county was either unavailable or unknown. Although a majority of BH providers directly provided continuing or aftercare services, one quarter referred elsewhere for these services, and in 10% of jurisdictions these services were unavailable or unknown to the providers. Both recovery support and continuing care are critical components for reducing relapse to substance use problems and recidivism among youth who have been involved in the JJ system (National Institute on Drug Abuse, 2014). The lack of access to these services within JJ systems of care indicates a risk of attrition of youth from the services cascade. Innovative models for delivery of recovery support services for youth include mHealth interventions using text-messaging prompts and supportive messages, which have shown promising outcomes with youth in substance use treatment (Dennis et al. 2015; Gonzales et al., 2014) and primary care (Shrier et al., 2018). Incorporating these interventions within BH services for JJ-involved youth may be especially warranted.

Consistent with the cascade model of service delivery to JJ-involved offenders, the findings suggest that many CS and BH providers have established mechanisms for referral, information exchange, and collaboration. One quarter of the paired CS and BH provider groups were classified as both “high” on indices of cross-system interactions and quality of services provision. However, close to two fifths of the paired CS-BH providers were classified as “low” on both the indices of cross-system interactions and quality of services provision, with the remainder falling into mixed classifications of high/low. It is noteworthy that the two groups with “high” CS ratings on collaboration and interactions (with BH either high or low), had the highest overall collaboration scores, demonstrating the value of CS leadership in these relationships. Other research examining the provision of BH services for youth involved in both the JJ and child welfare systems found that having a single agency accountable for youth care, along with inter-agency sharing of administrative data, increased the odds of youth receiving BH services (Chuang & Wells, 2010). Others have argued that a holistic understanding of youth’s social/environmental context and the involvement of families and community services are needed to minimize the adoption of “system-centric” approaches to assessing the needs of youth, which lead to a skewed understanding of their service needs (Maschi et al. 2008). Hence, efforts aimed at improving BH services provision to JJ-involved youth require both strong CS leadership and the adoption of a multisystemic approach to understanding and meeting their needs.

### Limitations

Several limitations of the study should be acknowledged. For example, many programs did not have access to information on the BH needs of youth they served, and among those that had such information, there was a wide range of measures and definitions used to assess BH status and needs. The current study included no internal measures to identify the background, knowledge, training, and other characteristics of respondents as well as the quality and validity of the clinical assessments. Admittedly, indices of cross-system interactions and quality of BH services relied on crude counts of activities performed, although we note that these measures met standards for internal validity.

## Conclusion

Despite these limitations, the study findings provide a barometer for progress in developing BH systems of care for JJ-involved youth, identifying gaps within these service systems, and highlighting areas where improvements are urgently needed. The findings show that although many elements in a cascade model of BH services for JJ-involved youth have been implemented through cross-system referrals and collaboration between CS and BH providers, there are several underdeveloped areas and potential for attrition across the service cascade. In particular, greater attention is needed to providing services that address the needs of youth with higher levels of severity, aftercare services, and recovery support. Future research should aim to develop interventions to address these identified gaps within JJ systems of care, as well as examine the relationship between systems-of-care characteristics and youth outcomes, such as recidivism.

## Additional file

Additional file 1: “Sources for JJ-TRIALS survey items,” provides a list of all sources for items used in the JJ-TRIALS national survey. (DOCX 29 kb)

## Data Availability

The datasets used and/or analyzed during the current study are available from the corresponding author on reasonable request.

## Abbreviations

BH: Behavioral health
CS: Community supervision
EBP: Evidence-based practice
FTE: Full-time equivalents
HIV: Human immunodeficiency virus
JJ: Juvenile justice
JJS: Juvenile justice system
JJ-TRIALS: Juvenile Justice-Translational Research on Interventions for Adolescents in the Legal System
OR: Odds ratio
STIs: Sexually transmitted infections

## References

[CR1] Abram KM, Choe JY, Washburn JJ, Teplin LA, King DC, Dulcan MK (2008). Suicidal ideation and behaviors among youths in juvenile detention. Journal of the American Academy of Child & Adolescent Psychiatry.

[CR2] Abram KM, Teplin LA, Charles DR, Longworth SL, McClelland GM, Dulcan MK (2004). Posttraumatic stress disorder and trauma in youth in juvenile detention. Archives of General Psychiatry.

[CR3] Abram KM, Teplin LA, McClelland GM, Dulcan MK (2003). Comorbid psychiatric disorders in youth in juvenile detention. Archives of General Psychiatry.

[CR4] Abram KM, Washburn JJ, Teplin LA, Emanuel KM, Romero EG, McClelland GM (2007). Posttraumatic stress disorder and psychiatric comorbidity among detained youths. Psychiatric Services.

[CR5] Abram KM, Zwecker NA, Welty LJ, Hershfield JA, Dulcan MK, Teplin LA (2015). Comorbidity and continuity of psychiatric disorders in youth after detention: A prospective longitudinal study. JAMA Psychiatry.

[CR6] Belenko S, Knight D, Wasserman GA, Dennis ML, Wiley T, Taxman FS, Oser C, Dembo R, Robertson AA, Sales J (2017). The juvenile justice behavioral health services cascade: A new framework for measuring unmet substance use treatment services needs among adolescent offenders. Journal of Substance Abuse Treatment.

[CR7] Champion DJ (2001). The juvenile justice system: Delinquency, processing, and the law.

[CR8] Chapman JF, Ford JD (2008). Relationships between suicide risk, traumatic experiences, and substance use among juvenile detainees. Archives of Suicide Research.

[CR9] Chuang E, Wells R (2010). The role of inter-agency collaboration in facilitating receipt of behavioral health services for youth involved with child welfare and juvenile justice. Children and Youth Services Review.

[CR10] Cocozza JJ, Skowyra KR, Shufelt JL (2010). Addressing the mental health needs of youth in contact with the juvenile justice system in system of care communities: An overview and summary of key issues.

[CR11] D'Amico EJ, Edelen MO, Miles JN, Morral AR (2008). The longitudinal association between substance use and delinquency among high-risk youth. Drug and Alcohol Dependence.

[CR12] Dennis ML, Scott CK, Funk RR, Nicholson L (2015). A pilot study to examine the feasibility and potential effectiveness of using smartphones to provide recovery support for adolescents. Substance Abuse.

[CR13] Dennis ML, White MK, Ives ML, Leukefeld CG, Gullotta TP, Staton-Tindall M (2009). Individual characteristics and needs associated with substance misuse of adolescents and young adults in addiction treatment.

[CR14] Donenberg GR, Emerson E, Mackesy-Amiti ME, Udell W (2015). HIV-risk with juvenile offenders on probation. Journal of Child and Family Studies.

[CR15] Epperson M, Wolff N, Morgan R, Fisher W, Frueh BC, Huening J (2011). *The next generation of behavioral health and criminal justice interventions: Improving outcomes by improving interventions.* New Brunswick, NJ: Center for Behavioral Health Services and Criminal Justice Research, Rutgers. The State University of new Jersey.

[CR16] Ford JD, Elhai JD, Connor DF, Frueh BC (2010). Polyvictimization and risk of posttraumatic, depressive, and substance use disorders and involvement in delinquency in a national sample of adolescents. Journal of Adolescent Health.

[CR17] Ford JD, Grasso DJ, Hawke J, Chapman JF (2013). Poly-victimization among juvenile justice-involved youths. Child Abuse and Neglect.

[CR18] Gonzales R, Ang A, Murphy DA, Gilk DC, Anglin DA (2014). Substance use recovery outcomes among a cohort of youth participating in a mobile-based texting aftercare pilot program. Journal of Substance Abuse Treatment.

[CR19] Grisso T (2004). Double jeopardy: Adolescent offenders with mental disorders.

[CR20] Grisso T, Underwood LA (2004). Screening and assessing mental health and substance use disorders among youth in the juvenile justice system.

[CR21] Howell JC, Kelly MR, Palmer J, Mangum RL (2004). Integrating child welfare, juvenile justice, and other agencies in a continuum of services. Child Welfare.

[CR22] Huizinga D, Loeber R, Thornberry T, Cothern L (2000). Co-occurrence of delinquency and other problem behaviors.

[CR23] Ives ML, Chan Y, Modisette KC, Dennis ML (2010). Characteristics, needs, services, and outcomes of youth in juvenile treatment drug courts as compared to adolescent outpatient treatment. Drug Court Review.

[CR24] Kaeble D, Glaze LE (2016). Correctional populations in the United States.

[CR25] Karnik NS, Soller M, Redlich A, Silverman M, Kraemer HC, Haapanen R, Steiner H (2009). Prevalence of and gender differences in psychiatric disorders among juvenile delinquents incarcerated for nine months. Psychiatric Services.

[CR26] King DC, Abram KM, Romero EG, Washburn JJ, Welty LJ, Teplin LA (2011). Childhood maltreatment and psychiatric disorders among detained youths. Psychiatric Services.

[CR27] Lansing AE, Washburn JJ, Abram KM, Thomas UC, Welty LJ, Teplin LA (2014). Cognitive and academic functioning of juvenile detainees: Implications for correctional populations and public health. Journal of Correctional Health Care.

[CR28] Lehman BWF (2009). H.K., Wexler, G., & Melnick. Organizational factors and collaboration and integration activities in criminal justice and drug abuse treatment agencies. Drug and Alcohol Dependence.

[CR29] Lipsey MW (2009). The primary factors that characterize effective interventions with juvenile offenders: A meta-analytic review. Victims and Offenders.

[CR30] Maschi T, Hatcher SS, Schwalbe CS, Rosato NS (2008). Mapping the social service pathways of youth to and through the juvenile justice system: A comprehensive review. Children and Youth Services Review.

[CR31] McCarty D, Chandler RK (2009). Understanding the importance of organizational and system variables on addiction treatment services within criminal justice settings. Drug and Alcohol Dependence.

[CR32] McCord J, Widom CS, Crowell NA (2001). Juvenile crime, juvenile justice.

[CR33] Mendel, R.A. (2011). *No place for kids: The case of reducing juvenile incarceration.* Baltimore, MD: The Annie E. Casey Foundation.

[CR34] National Institute on Drug Abuse. (2014). *Principles of adolescent substance use disorder treatment: A research-based guide*. (NIH Publication No. 14–7953). Bethesda, MD: National Institute on Drug Abuse.

[CR35] Odgers CL, Burnette ML, Chauhan P, Moretti MM, Reppucci ND (2005). Misdiagnosing the problem: Mental health profiles of incarcerated juveniles. Canadian Child and Adolescent Psychiatry Review.

[CR36] Osterlind SJ, Koller JR, Morris EF (2007). Incidence and practical issues of mental health for school aged youth in juvenile justice detention. Journal of Correctional Health Care.

[CR37] Pajer KA, Kelleher K, Gupta RA, Rolls J, Gardner W (2007). Psychiatric and mental health care policies in juvenile detention facilities. Journal of the American Academy of Child and Adolescent Psychiatry.

[CR38] Ramchand R, Morral AR, Becker K (2009). Seven-year life outcomes adolescent offenders in Los Angeles. American Journal of Public Health.

[CR39] Romero EG, Teplin LA, McClelland GM, Abram KM, Welty LJ, Washburn JJ (2007). A longitudinal study of the prevalence, development, and persistence of HIV/sexually transmitted infection risk behaviors in delinquent youth: Implications for health care in the community. Pediatrics.

[CR40] Sales JM, Wasserman G, Elkington KS, Lehman W, Gardner S, McReynolds L, Wiley T, Knudsen H (2018). Perceived importance of substance use prevention in juvenile justice: A multi-level analysis. Health and Justice.

[CR41] Schubert C, Mulvey EP (2014). Behavioral health problems, treatment, and outcomes in serious youthful offenders. Juvenile Justice Bulletin.

[CR42] Schubert C, Mulvey EP, Glasheen C (2011). Influence of mental health and substance abuse problems and criminogenic risk on outcomes in serious juvenile offenders. Journal of the American Academy of Child and Adolescent Psychiatry.

[CR43] Scott CK, Lurigio AJ, Dennis ML (2017). Judges’ perceptions of screening, assessment, prevention, and treatment for substance use, mental health, and HIV among juveniles on community supervision: Results of a national survey. Juvenile and Family Court Journal.

[CR44] Shrier LA, Burke PJ, Kells M, Scherer EA, Sarda V, Jonestrask C, Xuan Z, Harris SK (2018). Pilot randomized trial of MOMENT, a motivational counseling-plus-ecological momentary intervention to reduce marijuana use in youth. mHealth.

[CR45] Shufelt, J. L., & Cocozza, J. (2006). *Youth with mental health disorders in the juvenile justice system: Results from a multi-state prevalence study.* Delmar, NY: National Center for Mental Health and Juvenile Justice.

[CR46] Tapia M, McCoy H, Tucker L (2016). Suicidal ideation in juvenile arrestees: Exploring legal and temporal factors. Youth Violence and Juvenile Justice.

[CR47] Teplin LA, Abram KM, McClelland GM, Dulcan MK, Mericle AA (2002). Psychiatric disorders in youth in juvenile detention. Archives of General Psychiatry.

[CR48] Teplin LA, Mericle AA, McClelland GM, Abram KM (2003). HIV and AIDS risk behaviors in juvenile detainees: Implications for public health policy. American Journal of Public Health.

[CR49] Teplin LA, Stokes ML, McCoy KP, Abram KM, Byck GR (2015). Suicidal ideation and behavior in youth in the juvenile justice system: A review of the literature. Journal of Correctional Health Care.

[CR50] Thomas J, Gourley GK, Mele N (2005). The availability of behavioral health services for youth in the juvenile justice system. Journal of the American Psychiatric Nurses Association.

[CR51] Timmons-Mitchell J, Brown C, Schulz S, Webster S, Underwood L, Semple W (1997). Comparing the mental health needs of female and male incarcerated juvenile delinquents. Behavioral Sciences & the Law.

[CR52] Tolou-Shams M, Stewart A, Fasciano J, Brown LK (2009). A review of HIV prevention interventions for juvenile offenders. Journal of Pediatric Psychology.

[CR53] Trupin, E., & Boesky, L. (1999). *Working together for change: Co-occurring mental health and substance use disorders among youth involved in the juvenile justice system: Cross training, juvenile justice, mental health, and substance abuse.* Delmar, NY: The National GAINS Center.

[CR54] Underwood LA, Washington A (2016). Mental illness and juvenile offenders. International Journal of Environmental Research and Public Health.

[CR55] United States Census. (2012). 2010 Current Population Survey (CPS) Data File. https://www.census.gov/programs-surveys/cps.html. Accessed 8 February 2018.

[CR56] Wasserman GA, McReynolds LS, Lucas CP, Fisher P, Santos L (2002). The voice DISC-IV with incarcerated male youths: Prevalence of disorder. Journal of the American Academy of Child and Adolescent Psychiatry.

[CR57] Young DW, Dembo R, Henderson CE (2007). A national survey of substance abuse treatment for juvenile offenders. Journal of Substance Abuse Treatment.

