# Supplementary material for: Juvenile justice systems of care: results of a national survey of community supervision agencies and behavioral health providers on services provision and cross-system interactions
Source: Health Justice. 2019 Jun 14;7:11. doi: 10.1186/s40352-019-0093-x (PMC6717998; doi:10.1186/s40352-019-0093-x)
Supplement: Supplementary file 1 — “Sources for JJ-TRIALS survey items,” provides a list of all sources for items used in the JJ-TRIALS national survey. (DOCX 29 kb) [file 40352_2019_93_MOESM1_ESM.docx]

| **Instrument/Source** | **Reference** |
| --- | --- |
| Health Reform Readiness Index | Molfenter, T.D. (2014). Addiction treatment centers' progress in preparing for health care reform. *Journal of Substance Abuse Treatment*, 46(2), 158-164. |
| Inventory of Mental Health Services in Juvenile Justice Facilities | Goldstrom, I., Jaiquan, F., Henderson, M., Male, A., Manderscheid, R., Manderscheid, R.W., & Henderson, M.J. (2000). The availability of mental health services to young people in juvenile justice facilities: A national survey. In R.W. Manderscheid, & M.J. Henderson (Eds.), *Mental health, United States* (pp. 248-268). Rockville, MD: Substance Abuse and Mental Health Services Administration. |
| Juvenile Justice Assessment Planning, Referral, and Placement | Taxman, F.S., Henderson, C., Young, D., & Farrell, J. (2014). The impact of training interventions on organizational readiness to support innovations in juvenile justice offices*. Administration and Policy in Mental Health and Mental Health Services Research*, 41(2), 177-188. |
| National Center for Juvenile Justice | Knoll, C., & Sickmund, M. (2012). *Delinquency cases in juvenile court, 2009. Juvenile Offenders and Victims National Report Series*. Washington, DC: Office of Juvenile Justice and Delinquency Prevention. |
| National Criminal Justice Treatment Practices Survey | Taxman, F.S., Young, D.W., Wiersema, B., Rhodes, A., & Mitchell, S. (2007). The national criminal justice treatment practices survey: Multilevel survey methods and procedures. *Journal of Substance Abuse Treatment*, 32(3), 225-238. |
| National Survey of Substance Abuse Treatment Services | Substance Abuse and Mental Health Services Administration (SAMHSA). (2013). *National survey of substance abuse treatment services: 2013 survey*. Rockville, MD: Author. |
| Project Connect | Wasserman, G.A., McReynolds, L.S., Musabegovic, H., Whited, A.L., Keating, J.M., & Huo, Y. (2009). Evaluating Project Connect: Improving juvenile probationers’ mental health and substance use service access. *Administration and Policy in Mental Health and Mental Health Services Research*, 36(6), 393-405. |
| Recidivism Risk Workgroup | Harris, P.W., Lockwood, B., Mengers, L., & Stoodley, B.H. (2011). Measuring recidivism in juvenile corrections. *OJJDP Journal of Juvenile Justice*, 1(1), 1-11. |
| Risk Needs and Recidivism Models | Schwalbe, C.S. (2007). Risk assessment for juvenile justice: A meta-analysis. *Law and Human Behavior*, 31(5), 449-462. |
| Screening and Assessing Mental Health and Substance Use Disorders Among Youth in the Juvenile Justice System: A Resource Guide for Practitioners | Grisso, T., & Underwood, L.A. (2004). *Screening and assessing mental health and substance use disorders among youth in the juvenile justice system*. Washington, DC: Office of Juvenile Justice and Delinquency Prevention. |
| Standardized Program Evaluation | Lipsey, M.W. (2008). *The Arizona Standardized Program Evaluation Protocol (SPEP) for assessing the effectiveness of programs for juvenile probationers: SPEP ratings and relative recidivism reduction for the initial SPEP sample*. Nashville, TN: Center for Evaluation Research and Methodology. |
| State Regulation of Residential Facilities for Children with Mental Illness Survey | Iresys, H.T., Achman, L., & Takyi, A. (2006). *State regulation of residential facilities for children with mental illness*. DHHS Pub. No. (SMA) 06-4167. Rockville, MD: Center for Mental Health Services, Substance Abuse and Mental Health Services Administration.  Teich, J., & Ireys, H. (2007). A national survey of state licensing, regulating, and monitoring of residential facilities for children with mental illness. *Psychiatric Services*, 58(7), 991-998. |
| Strengthening Transnational Approaches to Reducing Reoffending | Koehler, J.A., Hamilton, L., & Lösel, F.A. (2013). Correctional treatment programs for young offenders in Europe: A survey of routine practice. *European Journal on Criminal Policy and Research*, 19(4), 387-400. |
| Survey of Recidivism Measures | Council of Juvenile Correctional Administrators. (2013). *Recidivism survey*. Braintree, MA: Author. |
| Survey of Youth in Residential Placement | Sedlak, A.J., & McPherson, K. (2010). *Survey of youth in residential placement: Youth’s needs and services*. Rockville, MD: Westat. |
| Survey on the Prevalence, Use, and Satisfaction of Case Management Systems in Probation and Parole | American Probation and Parole Association (APPA). (2013). *Survey on the prevalence, use, and satisfaction of case management systems (CMS) in probation and parole*. Lexington, KY: Author. |
